# Endometriosis, symptoms, and risk for depression and/or anxiety: a population-based retrospective study

**DOI:** 10.1186/s12905-025-04022-5

**Published:** 2025-10-08

**Authors:** Emma Goodwin, Helena Abreu do Valle, Amanda S. Nitschke, Joseph H. Puyat, Paul J. Yong, Gillian E. Hanley

**Affiliations:** 1 Department of Gynaecology and Obstetrics, Division of Gynaecologic Oncology, University of British Columbia, Vancouver, BC Canada; 2 School of Population and Public Health, University of British Columbia, Vancouver, BC Canada; 3 Vancouver General Hospital Research Pavilion, 590-828 West 10th Ave, Vancouver, BC Canada

**Keywords:** Endometriosis, Asymptomatic, Depression, Anxiety, Chronic pain, Inflammation

## Abstract

**Background:**

Endometriosis is a chronic and inflammatory condition that often presents with chronic pelvic pain, dysmenorrhea, and dyspareunia, thus having important effects on quality of life. There are two proposed hypotheses to describe the known association between endometriosis and depression and anxiety: (1) the disease hypothesis, where the inflammatory nature of endometriosis is driving increased risk for depression and anxiety; and (2) the pain hypothesis, where it is the painful symptoms underlying the increased risk. We aimed to shed further light on these two hypotheses by assessing the risk for depression and/or anxiety in three groups of patients pathologically assessed for endometriosis: symptomatic endometriosis patients (Symp Endo), symptomatic patients with no pathological endometriosis diagnosis (Symp No Endo), and asymptomatic endometriosis patients (Asymp Endo).

**Methods:**

This study included pathologically-confirmed endometriosis patients identified from the pathology records of Vancouver Coastal Health Authority between 2000 and 2008. These data were linked with population-based administrative health data for follow-up to 2017. Depression and anxiety were identified through diagnostics codes from health services use data and prescriptions for antidepressants. Bivariate analyses were performed to assess differences between groups. Cox proportional hazards models were run to generate hazard ratios for incident depression and/or anxiety between the groups.

**Results:**

There were 2729 people in Symp Endo, 585 in Symp No Endo, and 326 in Asymp Endo. Symp No Endo was more likely than Symp Endo to be visiting a physician for pelvic pain and to be taking prescription-level pain medications (*p* < 0.001). After adjusting for several covariates, Symp No Endo had a significantly higher risk of incident depression and/or anxiety (adjusted HR: 1.23, 95% CI: 1.06–1.41) compared to Symp Endo. There was no statistically significant difference in risk of depression and/or anxiety between Symp Endo and Asymp Endo (adjusted HR: 0.94, 95% CI: 0.76–1.17).

**Conclusions:**

These results point toward the pain focused hypotheses as Symp No Endo patients were at higher risk for depression/anxiety than the Symp Endo group. However, the results also suggest the disease hypothesis is at play, because the Symp Endo and Asymp Endo groups were at the same level of risk for depression/anxiety.

**Supplementary Information:**

The online version contains supplementary material available at 10.1186/s12905-025-04022-5.

## Background

Endometriosis is a chronic, estrogen-dependent, inflammatory condition characterized by the growth of endometrial-like tissue outside of the uterus [1]. It is estimated to affect approximately 10% of people assigned female at birth, with a rate of asymptomatic endometriosis, usually diagnosed in those having surgery for other indications, that may be as high as another 10% in the general population [1, 2]. Common symptoms include chronic pelvic pain, dysmenorrhea, dyspareunia, dyschezia, and dysuria. First-line treatments are usually hormonal medications, such as combined oral contraceptives or hormonal intrauterine devices (IUD), and/or prescription-level analgesics [1, 3]. Surgical treatment, including hysterectomy with or without bilateral salpingo-oophorectomy, may also be offered. Surgical treatment is normally used when hormonal medications are not appropriate. This may be due to desire of patient to conceive, hormonal medications are not tolerated well by the patient, or symptoms were not relieved by other treatments [4, 5].

Previous cohorts reported depression rates among people with endometriosis of approximately 15% and anxiety rates as high as 29%, compared with a l ifetime prevalence of 11.2% for depression and 8.7% for anxiety in the general population [6–11]. Thus, it is generally well accepted that endometriosis is associated with increased risk for depression and/or anxiety [12]. However, there is limited human literature examining what is driving the increased risk for depression and anxiety among endometriosis patients. Conflicting evidence from small cohort studies have shown both no difference in depression and anxiety between symptomatic and asymptomatic endometriosis patients and higher depression and anxiety scores in symptomatic endometriosis patients compared to those who are asymptomatic and healthy controls [13, 14]. 

There have been two hypotheses have been proposed for understanding this association between endometriosis and depression and/or anxiety [13]. The first is the pain-focused hypothesis, where the chronic and painful symptoms of endometriosis cause negative effects on mental health and quality of life. The second is the disease-focused hypothesis. Due to the inflammatory nature of endometriosis, cytokine levels can be disrupted systemically, and subsequent effects are observed in various parts of the body. These changes can affect the perception of depression and distress, leading to acute and chronic anxiety and depression. These conditions can then also affect the immune system, leading to a cyclic effect. There is some support for this hypothesis from the animal literature showing that mice exhibit more anxiety- and depression-like behaviours following induction of endometriosis compared to control mice; however, this work needs to be examined in human studies [15]. This has added to a growing body of literature that is acknowledging endometriosis as a systemic condition that is not isolated to the pelvis [16]. 

Herein, we aim to determine whether, and how, risk for depression and/or anxiety differs across three groups with differing endometriosis symptomology and pathological diagnoses. This will shed light on the mechanism driving the association between endometriosis and mental illness using a cohort of population-based administrative data. With these data, we will be able to examine a large sample of endometriosis and pelvic pain patients over time. Additionally, we have access to potential confounders and modifiers in this dataset.

## Methods

### Cohort

We conducted a population-based retrospective cohort study of all people with endometriosis in the final diagnosis in their pathology report in the pathology database of Vancouver Coastal Health (population ~ 1 million) in British Columbia (BC), Canada. We used the search terms ‘endometriosis’, ‘endometrioma’ and/or ‘endometriotic cyst’ and examined all pathology reports with those search terms between the years 2000 and 2008. These data were linked to population-based administrative datasets, containing information on hospitalizations and physician visits, and BC PharmaNet, which contains records of every prescription dispensed in an outpatient setting, between 1998 and 2017 [17–24]. Ethical approval was obtained from the University of British Columbia Clinical Research Ethics Board. All inferences, opinions, and conclusions are those of the authors, and do not reflect the opinions or policies of the Data Stewards. Approval by the Ethics Board and the BC Data stewards for use of deidentified administrative data files includes a waiver of informed consent from participants. This research was conducted in accordance with the Declaration of Helsinki.

### Inclusion and exclusion criteria

All people undergoing surgery in Vancouver Coastal Health authority between 2000 and 2008 with a subsequent pathology report that contained any of the search terms listed above in the final diagnosis were included. If the search term referenced endometriosis not being present, for example if the sample was negative for endometriosis, these instances were included as negative cases. The surgery that resulted in inclusion in the cohort is referred to as their index surgery, which is the first surgery where the patient was diagnosed with endometriosis, or the first surgery that included our search terms in the subsequent pathology report during the study period for patients who were never diagnosed with endometriosis. Patients were excluded if: (1) their age was > 80 years or < 15 years; (2) they were diagnosed with a gynecologic malignancy at any point in our study period; (3) they had any history of schizophrenia, manic disorder, bipolar disorder, or psychosis; or (4) they had depression and/or anxiety diagnostic codes (as defined below), or had filled a prescription for serotonin reuptake inhibitor (SRI) prescription, in the two years prior to their index surgery. Criteria four was used to identify incident diagnoses of depression and/or anxiety after the index surgery.

### Exposure identification

First, we looked at each patient’s history of physician visits for endometriosis or pelvic pain, or a previous endometriosis surgery. Second, we examined the indication for the surgery that resulted in introduction to our cohort (which we call the index surgery) to determine whether endometriosis was suspected. Third, based on the pathology report that accompanied their index surgery, we could determine whether or not each person was diagnosed with pathologically-confirmed endometriosis. We then created the following three groups based on their symptomology and final diagnosis in the pathology report from their index surgery: Symp Endo: People suspected to have endometriosis with a history of physician visits for endometriosis or pelvic pain, or previous endometriosis surgery, with endometriosis pathologically confirmed at the index surgery (symptomatic endometriosis); Symp No Endo: People with suspected endometriosis at index surgery indication or a history of physician visits for endometriosis or pelvic pain, but no pathological endometriosis in their index surgery (symptomatic with no endometriosis); and Asymp Endo: People who had no indication of endometriosis (their indication for surgery was something unrelated, e.g. fibroids) and no history of physician visits for endometriosis or pelvic pain for two years prior to their index surgery, but pathological endometriosis was confirmed during their index surgery (asymptomatic endometriosis).

### Outcome identification

Physician visits and hospital data were examined to look for depression and/or anxiety International Classification of Diseases (ICD) diagnostic codes following their index surgery (Supplementary Table 1). We present the data with depression and/or anxiety as a composite outcome because of the presence of a diagnostic code (ICD-9 code 50B) that is specific to British Columbia that groups the two conditions together. A person was defined as having depression and/or anxiety if they met one of these criteria following their index surgery: (1) they had two diagnostic codes for depression and/or anxiety in one year, at least 30 days apart; or (2) they had one diagnostic code for depression and/or anxiety and a SRI prescription dispensed. This method to identify depression and/or anxiety has previously been validated in these databases and found to have high rates of accuracy in identifying psychiatric conditions [25, 26]

### Covariates

We adjusted for patient age and neighborhood income quintile. We include neighborhood income quintile as it can be a non-medical determinant of health, and/or an enabling factor for access to care, and has been validated to approximate individual level income [27]. We also controlled for hysterectomy at index surgery, bilateral oophorectomy (BO), bilateral salpingoophorectomy (BSO), or second unilateral oophorectomy (UO) or unilateral salpingoophorectomy (USO) prior to or at index surgery, and other diagnoses at pathology report (endometrial hyperplasia, adenomyosis, or fibroids).

### Statistical analysis

Patient characteristics, gynecologic conditions, and procedures occurring during the index surgery were compared using chi-squared test for dichotomous and categorical variables and t-tests for continuous, normally distributed variables to look for statistically significant differences. Effect sizes were determined with Cohen’s D or Cramer’s V. We also compared the physician visits and medications prescribed in the two years prior to index surgery across the three groups.

We compared the groups using Cox proportional hazards model, with Symp Endo as the reference group, and the resulting hazard ratios (HRs) acted as a proxy risk for depression and/or anxiety. The proportional hazards assumptions were assessed after fitting all the models using Schoenfeld residuals for nonzero slope. We found no evidence that this assumption was violated.

In the models, the date of diagnosis with depression and/or anxiety was defined as the date of the first relevant diagnostic code for persons who meet the criteria outlined above. People were censored upon death or if they were registered in the province’s universal health insurance for less than 200 days in a year, as we could not trust we had complete data capture for these people.

### Sensitivity analysis

Endometriosis, an estrogen-dependent clinical syndrome, is traditionally viewed as a premenopausal condition that naturally resolves after menopause or remains in a dormant state [28]. Thus, we hypothesize that any observed relationships are less likely to be consistent in menopausal populations. To determine how menopausal status may have altered the association between the endometriosis groups and depression and/or anxiety following surgery, we conducted a sensitivity analysis removing anyone who was in menopause in our cohort. This included patients who were age 50 or older, had a prior BO, BSO, second UO, second USO, or any of those procedures during their index surgery.

Further, as infertility is common in endometriosis patients, we isolated people undergoing their index surgery for infertility, but had no history or clinical indication of pain, in the form of physician visits for pelvic pain or endometriosis in the two years prior to the index surgery and where pain was not an indication for the index surgery. We also removed any individuals who had a hysterectomy or those over the age of 50 as their infertility is unrelated to their endometriosis. This allowed us to examine the risk for depression and/or anxiety in an endometriosis group experiencing infertility but without a history of pain in comparison to the group with symptomatic endometriosis (Symp Endo).

## Results

### Cohort

**Fig. 1:**
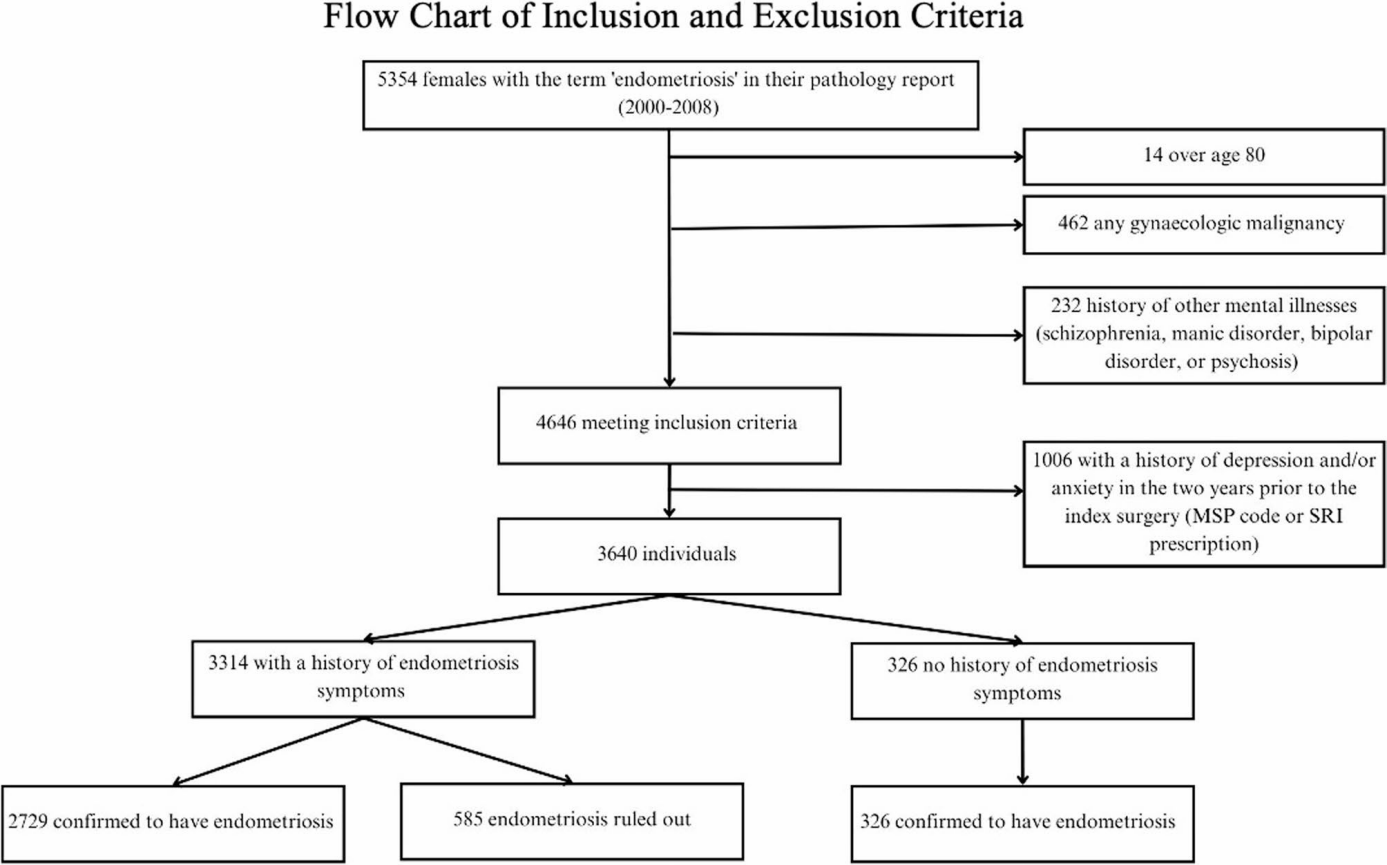
Flowchart of cohort inclusion and exclusion criteria

The cohort consisted of 5354 females with our search terms in their pathology report between 2000 and 2008. Once exclusion criteria were applied, the cohort totalled 3640 people. There were 2729 in Symp Endo (symptomatic with pathologically confirmed endometriosis), 585 in Symp No Endo (symptomatic without pathologically confirmed endometriosis), and 326 in Asymp Endo (asymptomatic with pathologically confirmed endometriosis; (Fig. 1).

Demographics and surgical descriptions are displayed in Table 1. There was no significant difference between the mean age of Symp Endo (38.2 years) and Symp No Endo (38.4 years). Asymp Endo was significantly older than Symp Endo, with an average age of 46.9 years (*p* < 0.001) and significantly more of Asymp Endo was menopausal (*p* < 0.001).

**Table 1:** Demographic, surgical, and diagnostic comparison of three exposure groups

Characteristics	Symp Endo (Symptomatic With Endometriosis, *N* = 2729)	Symp No Endo (Symptomatic Without Endometriosis, *N* = 585)	*P*-Value	Effect Size	Asymp Endo (Asymptomatic With Endometriosis, *N* = 326)	*P*-Value	Effect Size
Age (Mean (SD))	38.2 (8.76)	38.4 (10.0)	0.695	0.08	46.9 (9.68)	< 0.001	0.20
Income quintile							
1	557 (20.4%)	94 (16.1%)	0.010	0.06	64 (19.6%)	0.417	0.04
2	577 (21.1%)	122 (20.9%)			59 (18.1%)		
3	510 (18.7%)	120 (20.5%)			56 (17.2%)		
4	546 (20.0%)	148 (25.3%)			73 (22.4%)		
5	536 (19.6%)	101 (17.3%)			74 (22.7%)		
Missing	<=5	0 (0%)			0 (0%)		
Year of pathology report							
Median [min, max]*	2005 [2000, 2008]	2005 [2000, 2008]	0.16	0.08	2005 [2000, 2008]	0.16	0.09
Premenopausal age at surgery (proxy using age < 50)	2505 (91.8%)	523 (89.4%)	0.0739	0.03	211 (64.7%)	< 0.001	0.26
Postmenopausal age at surgery (proxy using age > = 50)	224 (8.2%)	62 (10.6%)	0.0739	0.03	115 (35.3%)	< 0.001	0.26
Procedures performed during the index surgery							
Hysterectomy	611 (22.4%)	118 (20.2%)	0.263	0.02	168 (51.5%)	< 0.001	0.21
Salpingectomy with ovarian conservation	61 (2.2%)	14 (2.4%)	0.936	0.001	10 (3.1%)	0.454	0.01
UO/USO	536 (19.6%)	73 (12.5%)	< 0.001	0.07	54 (16.6%)	0.209	0.02
BO/BSO/second UO/USO	332 (12.2%)	40 (6.8%)	< 0.001		81 (24.8%)	< 0.001	0.11
Biopsy	249 (9.1%)	105 (17.9%)	< 0.001	0.11	13 (4.0%)	0.003	0.05
Adhesiolysis	714 (26.2%)	135 (23.1%)	0.134	0.03	19 (5.8%)	< 0.001	0.15
Excision	764 (28.0%)	136 (23.2%)	0.022	0.04	21 (6.4%)	< 0.001	0.15
Ablation	421 (15.4%)	84 (14.4%)	0.556	0.01	<=5	< 0.001	0.13
Other	1046 (38.3%)	167 (28.5%)	< 0.001	0.08	32 (9.8%)	< 0.001	0.18
Surgical approach							
Laparoscopic	666 (24.4%)	148 (25.3%)	0.687	0.007	14 (4.3%)	< 0.001	0.15
Abdominal	478 (17.5%)	59 (10.1%)	< 0.001	0.08	72 (22.1%)	0.051	0.04
Vaginal	220 (8.1%)	61 (10.4%)	0.075	0.03	28 (8.6%)	0.824	0.004
Laparoscopic/vaginal combination	121 (4.4%)	41 (7.0%)	0.012	0.04	11 (3.4%)	0.456	0.01
Missing	485 (17.8%)	76 (13.0%)	0.006	0.05	84 (25.8%)	< 0.001	0.06
Clinical Indication for surgery from pathology report							
Endometrioma	353 (12.9%)	21 (3.6%)	< 0.001	0.11	0 (0%)	< 0.001	0.12
Other endometriosis	883 (32.4%)	194 (33.2%)	0.742	0.01	0 (0%)	< 0.001	0.22
Mass/suspected cancer	466 (17.1%)	44 (7.5%)	< 0.001	0.10	57 (17.5%)	0.914	0.002
Pain	431 (15.8%)	186 (31.8%)	< 0.001	0.16	0 (0%)	< 0.001	0.14
Infertility	236 (8.6%)	43 (7.4%)	0.345	0.02	<=5	< 0.001	0.08
Cyst	478 (17.5%)	72 (12.3%)	0.003	0.05	35 (10.7%)	0.003	0.05
Adenomyosis	14 (0.5%)	<=5	0.489	0.01	<=5	1	0.000
Fibroids	287 (10.5%)	56 (9.6%)	0.545	0.01	81 (24.8%)	< 0.001	0.13
Surgical history							
Prior BO/BSO, second UO/USO	<=5	<=5	0.793	0.004	<=5	1	0.000
Endometriosis surgery > 45 days before path report	318 (11.7%)	76 (13.0%)	0.402	0.01	0 (0%)	< 0.001	0.12
Pathologically diagnosed conditions							
Endometriosis	2729 (100%)	0 (0%)	< 0.001	1.0	326 (100%)	NA	NA
Endometrioma/endometriotic cyst	1108 (40.6%)	0 (0%)	< 0.001	0.33	59 (18.1%)	< 0.001	0.14
Endometriosis in ovary - non-endometrioma/endometriotic cyst	457 (16.7%)	0 (0%)	< 0.001	0.18	46 (14.1%)	0.257	0.02
Other endometriosis in the pelvis	1814 (66.5%)	0 (0%)	< 0.001	0.51	254 (77.9%)	< 0.001	0.07
Other endometriosis outside the pelvis	102 (3.7%)	0 (0%)	< 0.001	0.08	16 (4.9%)	0.376	0.02
Endometrial hyperplasia	32 (1.2%)	6 (1.0%)	0.929	0.001	11 (3.4%)	0.003	0.05
Adenomyosis	290 (10.6%)	47 (8.0%)	0.071	0.03	59 (18.1%)	< 0.001	0.07
Fibroids	520 (19.1%)	77 (13.2%)	< 0.001	0.06	149 (45.7%)	< 0.001	0.20

*SD* Standard Deviation* UO* Unilateral oophorectomy,* USO* Unilateral Salpingoophorectomy,* BO* Bilateral Oophorectomy,* BSO* Bilateral Salpingoophorectomy

When examining the index surgery procedures, Symp No Endo was less likely to have undergone a procedure including oophorectomy (USO, UO, or BSO, BO), biopsy, or other procedure compared to Symp Endo (*p* < 0.001). Contrastingly, Asymp Endo was more likely to have undergone a hysterectomy, procedures including oophorectomy, adhesiolysis, excision, ablation, and other procedure, compared to Symp Endo (*p* < 0.001). In terms of surgical approach, Symp No Endo was less likely to have had an abdominal approach. Asymp Endo was less likely to have had a laparoscopic approach.

The clinical indications for surgery were also different among the groups. Symp No Endo was more likely than Symp Endo to have their indication be pain (*p* < 0.001), but less likely to have mass/suspected cancer, endometrioma, and cyst (*p* < 0.05). Asymp Endo were less likely to have a clinical indication for infertility or cysts, and more likely to have fibroids as their indication compared to Symp Endo (*p* < 0.05).

Finally, when examining gynecologic diagnoses in the pathology report, Symp No Endo were less likely than Symp Endo to be diagnosed with fibroids (*p* < 0.001). Asymp Endo was less likely than Symp Endo to be diagnosed with endometrioma/endometriotic cysts, but more likely to have other endometriosis in the pelvis, endometrial hyperplasia, adenomyosis, and fibroids (*p* < 0.001).

Table 2 illustrates the differences between groups in the two years before the index surgery. Symp No Endo was significantly more likely than Symp Endo to have visited a physician for pelvic pain related concerns (*p* < 0.001). Symp No Endo was also more likely to be prescribed hormonal contraceptives, local estrogens, and antidepressants (*p* < 0.05). Asymp Endo was less likely than Symp Endo to be prescribed hormonal contraceptives and GnRH agonists (*p* < 0.05). Asymp Endo was significantly more likely to have a prescription for systemic estrogens and local estrogens (*p* < 0.05).

**Table 2:** Differences in health services use between groups in the two years before the index surgery

**Outcomes**	Symp Endo (Symptomatic With Endometriosis, *N* = 2729)	Symp No Endo (Symptomatic Without Endometriosis, *N* = 585)	*P*-Value	Effect Size	Asymp Endo (Asymptomatic With Endometriosis, *N* = 326)	*P*-Value	Effect size
Physician visits							
* For Pelvic Pain*							
At least one visit	1414 (51.8%)	432 (73.8%)	< 0.001	0.17	0 (0%)	< 0.001	0.32
At least two visits in one year (30 days apart)	683 (25.0%)	251 (42.9%)	< 0.001	0.15	0 (0%)	< 0.001	0.18
* For Endometriosis*							
At least one visit	917 (33.6%)	183 (31.3%)	0.302	0.02	0 (0%)	< 0.001	0.23
At least two visits in one year (30 days apart)	412 (15.1%)	85 (14.5%)	0.776	0.005	0 (0%)	< 0.001	0.13
Hormonal medications (any prescription)							
* Estrogens and Progestogens combined*							
Hormonal contraceptives	888 (32.5%)	224 (38.3%)	0.0087	0.05	73 (22.4%)	< 0.001	0.07
Hormone replacement therapy	91 (3.3%)	29 (5.0%)	0.0743	0.03	15 (4.6%)	0.307	0.02
* Other*							
Systemic estrogens	51 (1.9%)	13 (2.2%)	0.691	0.01	14 (4.3%)	0.008	0.05
Progestogens	228 (8.4%)	61 (10.4%)	0.126	0.03	29 (8.9%)	0.82	0.004
Local estrogens	46 (1.7%)	17 (2.9%)	0.0727	0.03	16 (4.9%)	< 0.001	0.07
GnRH agonists	232 (8.5%)	41 (7.0%)	0.268	0.02	15 (4.6%)	0.020	0.04
Prescription-level analgesics							
* NSAIDs*							
Any prescription	1492 (54.7%)	349 (59.7%)	0.031	0.04	145 (44.5%)	< 0.001	0.06
Number of prescriptions							
Median [Min, Max]	2.00 [1.00, 73.0]	2.00 [1.00, 45.0]	< 0.001	0.08	2.00 [1.00, 39.0]	0.768	0.01
Number of days dispensed							
Median [Min, Max]	23.0 [2.00, 2320]	30.0 [1.00, 1200]	< 0.001	0.08	25.0 [2.00, 1520]	0.451	0.02
* Opioids*							
Any prescription	1661 (60.9%)	412 (70.4%)	< 0.001	0.07	174 (53.4%)	0.0108	0.05
Number of prescriptions							
Median [Min, Max]	2.00 [1.00, 363]	2.00 [1.00, 520]	< 0.001	0.16	1.50 [1.00, 47.0]	0.209	0.03
Number of days dispensed							
Median [Min, Max]	8.00 [1.00, 3610]	13.0 [1.00, 3140]	< 0.001	0.16	7.00 [1.00, 1100]	0.302	0.02
Psychotropics (any prescription)							
Anticonvulsants	49 (1.8%)	20 (3.4%)	0.0195	0.04	7 (2.1%)	0.819	0.004
Antidepressants	138 (5.1%)	45 (7.7%)	0.015	0.04	10 (3.1%)	0.149	0.03
SRIs	0 (0%)	0 (0%)	NA	NA	0 (0%)	NA	NA

*GnRH* Gonadotropin releasing hormone,* NSAID* Non-steroidal anti-inflammatory drugs* SRI* Serotonin reuptake inhibitor

Symp Endo (symptomatic endometriosis) was compared to Symp No Endo (symptomatic without endometriosis). Finding higher depression/anxiety risk in this comparison would be interpreted as support for the disease-focused hypothesis (because Symp Endo has the contributing factor of endometriosis in addition to pain). Alternatively, finding similar depression/anxiety in Symp No Endo compared to Symp Endo could suggest the pain-focused hypothesis is at play. Second, Asymp Endo (asymptomatic endometriosis) was compared to Symp Endo (symptomatic endometriosis). Finding higher depression/anxiety risk in this comparison would support the pain-focused hypothesis (because depression/anxiety is higher in symptomatic people with endometriosis). Finding a similar risk for depression/anxiety between Symp Endo and Asymp Endo could alternatively point to the disease-focused hypothesis (because depression/anxiety is similar in people with endometriosis regardless of pain status).

**Fig. 2:**
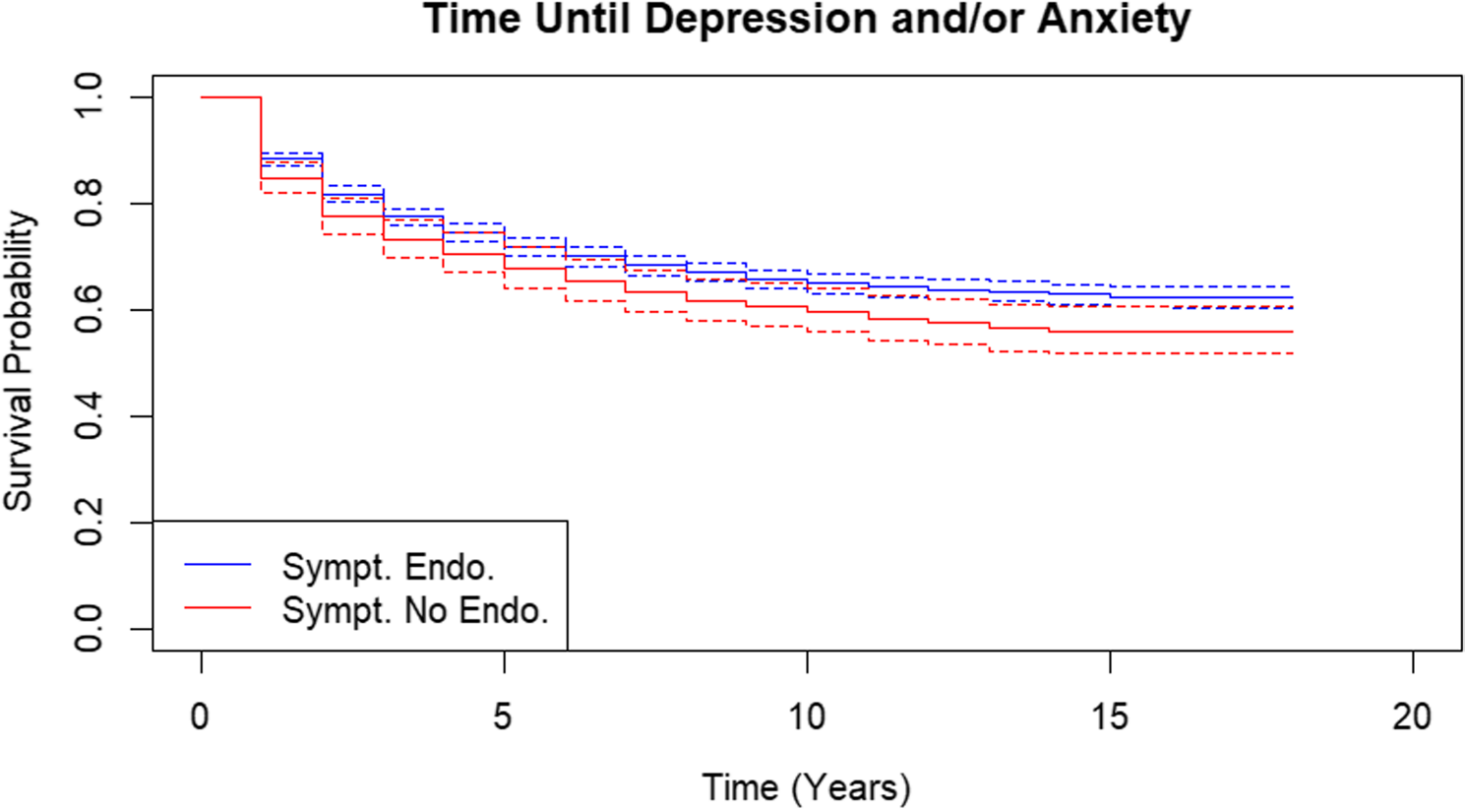
Kaplan-Meier curves for incidence of depression and/or anxiety in symptomatic endometriosis patients and symptomatic patients without endometriosis

**Fig. 3:**
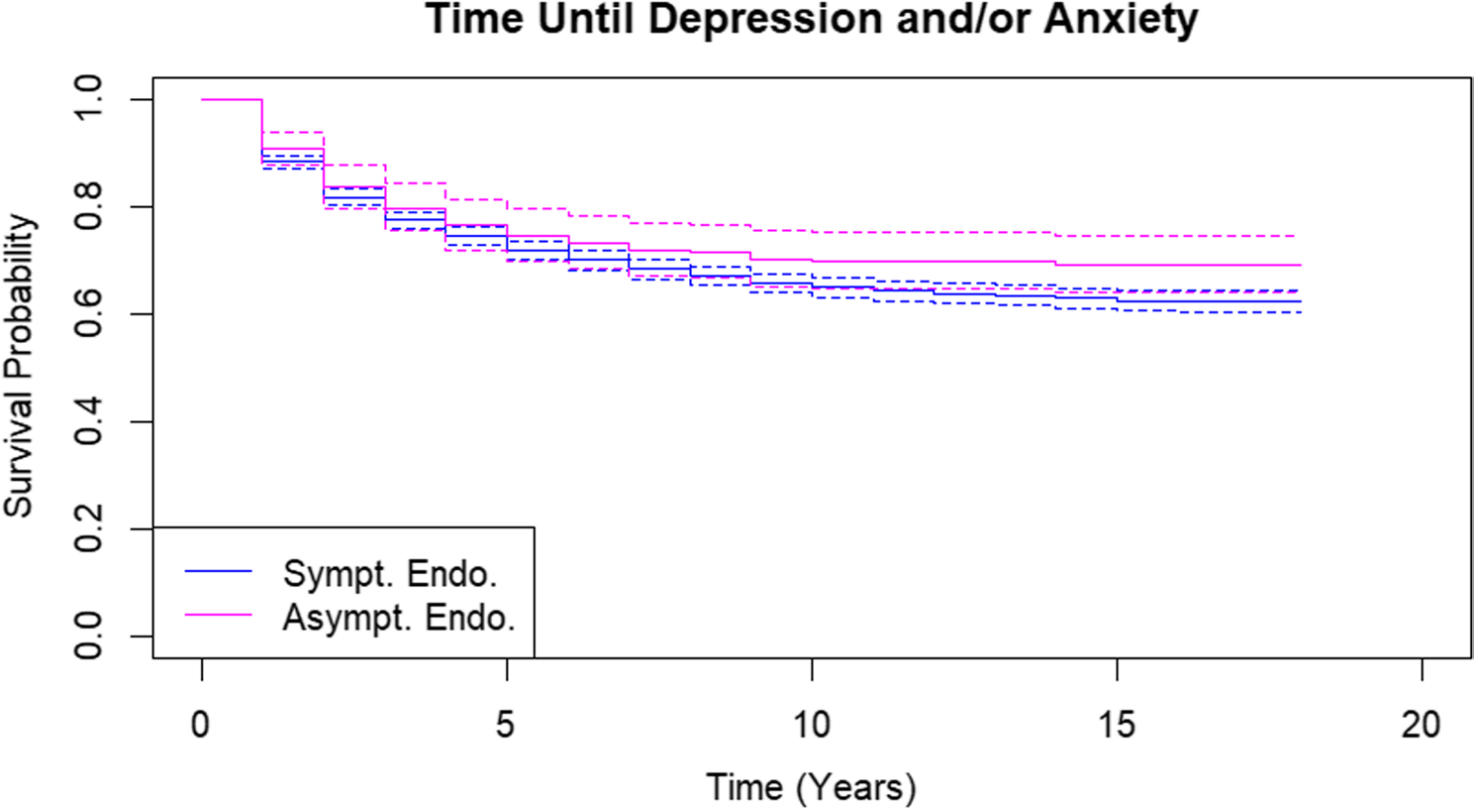
Kaplan-Meier curves for incidence of depression and/or anxiety in symptomatic endometriosis patients and asymptomatic endometriosis patients

Kaplan Meier curves show that Symp No Endo was more likely to be diagnosed with depression and/or anxiety than Symp Endo (Figure 2). The crude HR also showed that Symp No Endo had a 23% increased risk of depression and/or anxiety (Table 3, HR: 1.23, 95% CI: 1.06-1.41) compared to Symp Endo. There was no statistically significant difference between Kaplan Meier curves for Asymp Endo and Symp Endo (Figure 3). The crude HR for Asymp Endo was below 1 but not statistically significant (HR: 0.82, 95% CI: 0.66-1.01). Once adjusting for covariates, the HR for Symp No Endo remained the same at 23% (1.23, 95% CI: 1.06-1.41) and the HR for Asymp Endo was 0.94 (95% CI: 0.76-1.17).

**Table 3:** Unadjusted and adjusted hazard ratios for incidence of depression and/or anxiety after the index surgery

	Symp Endo (Symptomatic With Endometriosis)	Symp No Endo (Symptomatic Without Endometriosis)	Asymp Endo (Asymptomatic With Endometriosis)
N	2729	585	326
Mean Follow-up Time (years)	8.72	7.74	8.99
Number of Events	969	239	96
Crude hazard ratio (95% CI)	Ref	1.23 (1.06–1.41)	0.82 (0.66–1.01)
Adjusted hazard ratio^a^ (95% CI)	Ref	1.23 (1.06–1.41)	0.94 (0.76–1.17)

*HR *Hazard ratio,*CI*Confidence Interval

^a^Age, income quintile, hysterectomy at index, bilateral salpingoophorectomy/bilateral oophorectomy or second unilateral salpingoophorectomy/unilateral oophorectomy prior to or at index surgery, and other diagnoses at path report (endometrial hyperplasia, adenomyosis, or fibroids)

After removing individuals over age 50 at index surgery (Table 4, n=680 removed), our results were similar (Symp No Endo aHR=1.24 (95%CI: 1.06-1.45; Asymp Endo aHR=1.06 (95%CI: 0.81-1.39)). For the infertility sensitivity analysis, additional group comparisons are provided in Supplementary Table 2. This sensitivity analysis revealed that the infertility group had a crude HR of 0.75 (95% CI: 0.57-1.00). The adjusted HR revealed a 26% lower risk for depression and/or anxiety in this group (aHR=0.74 (95% CI 0.55-0.98) (Supplementary Table 3).

**Table 4:** Sensitivity analysis unadjusted and adjusted hazard ratios for incidence of depression and/or anxiety after the index surgery. To determine how menopausal status May have altered the association between the endometriosis groups and depression and/or anxiety following surgery, this sensitivity analysis excluded patients who were age 50 or older, had a prior BO, BSO, second UO, second USO, or any of those procedures during their index surgery

	Symp Endo (Symptomatic With Endometriosis)	Symp No Endo (Symptomatic Without Endometriosis)	Asymp Endo (Asymptomatic With Endometriosis)
N	2282	500	178
Mean Follow-up Time (years)	8.62	7.50	7.61
Number of Events	717	181	41
Crude hazard ratio (95% CI)	Ref	1.24 (1.06–1.44)	0.96 (0.74–1.25)
Adjusted hazard ratio^a^ (95% CI)	Ref	1.23 (1.06–1.44)	1.06 (0.81–1.26)

Group Criteria: menopausal (age or surgical) individuals removed from cohort (removed 680 people, N = 2, 282)

*HR *Hazard ratio,* CI *Confidence Interval

^a^Variables adjusted for: Age, income quintile, and other diagnoses at path report (adenomyosis, or fibroids)

## Discussion

### Main findings

We observed no statistically significant difference in risk of depression and/or anxiety in asymptomatic endometriosis patients (Asymp Endo) compared to symptomatic endometriosis patients (Symp Endo) (aHR = 1.06 (95%CI 0.81-1.26). This suggests that the presence of endometriosis symptoms was not significantly altering the risk for depression and/or anxiety between these groups, which is in line with the disease-focused hypothesis. We also found that the symptomatic group experiencing pelvic pain, who did not have endometriosis tissue identified in their pathology report (Symp No Endo), were at a 23% increased risk for depression and/or anxiety when compared to a symptomatic, pathologically-confirmed endometriosis group (Symp Endo). In the group with infertility as an indication for index surgery and no history of pelvic pain, a significantly lower (4%) risk of depression and/or anxiety was observed. These results suggest that the pain-focused hypothesis is contributing to this association. Thus, taken together, our results suggest that both mechanisms may be operating in endometriosis patients. It is thought that, in the majority of cases, the decreased estrogen levels associated with the menopausal period cause endometriosis symptoms to resolve [29]. Thus, it is possible that the disease model may be more relevant in premenopausal women, however this requires more study. The nature of endometriosis tissue itself post-menopause, and how it may be affected by estrogen release from adipose tissue or hormonal therapies, is not well understood [30]. While the results of our sensitivity analysis suggested that results were similar when we excluded people over age 50, it is difficult to comment on how the disease-focused hypothesis may be affecting risk for depression and/or anxiety in post-menopausal endometriosis patients, and more research is needed in this area.

Research has shown that individuals with similar chronic and inflammatory conditions (such as inflammatory bowel disease), report higher prevalence of depression and anxiety [31–33]. This is consistent with our finding that Asymp Endo and Symp Endo were at similar risk for depression and/or anxiety, suggesting there is an inflammatory effect possibly contributing to risk. Finally, our findings from our infertility sensitivity analysis are consistent with what was previously reported by Warchenza et al. They reported no correlation between prevalence of infertility and incidence of depression in their endometriosis cohort [8].

Our finding that, compared to Symp Endo, Symp No Endo patients were at a higher risk for depression and/or anxiety conflicts with previous studies that have shown no difference in the incidence of depression in those with pelvic pain, with and without endometriosis [8]. There are several possible explanations for this. First, based on the results showing that Symp No Endo had a higher likelihood of visiting a physician for pelvic pain, using hormonal contraceptives, NSAIDs, opioids, and antidepressants, it is possible that Symp No Endo was more symptomatic and experiencing more pain than Symp Endo, which would further point toward the pain-focused hypothesis. However, as we have no direct measure of pain from study participants, we cannot rule out that something else may be driving these findings. Only a small proportion of Symp No Endo were diagnosed with any of the conditions we were able to capture in the pathology report (0.9% with endometrial hyperplasia, 8.2% adenomyosis, and 12.9% with fibroids). While we could not capture every condition that may have been diagnosed following surgery, it’s possible that the lack of gynecologic diagnosis following the index surgery, may have resulted in more distress about their continuing symptoms, which increased risk for depression and/or anxiety. It is also possible that experiencing significant pain while also experiencing diagnostic uncertainty is, itself, a risk factor for anxiety and/or depression—a hypothesis that would warrant further study.

Our results support the clinical shift to treating endometriosis as a systemic, rather than a pelvic, condition. Both the disease- and pain-focused hypotheses should be considered in future research on the relationship between endometriosis and mental health. Further research should focus on understanding the nature of postmenopausal endometriosis, as well as how this relates to risk for depression and/or anxiety.

### Strengths and limitations

This study uses a large, pathologically-confirmed endometriosis cohort, which includes an asymptomatic group of endometriosis patients—a group that is very difficult to study. Additionally, we have a long follow up period for all three Groups presented. Our limitations include that population-based administrative data in BC are missing some potentially important covariates that are not routinely collected (i.e. body mass index, ethnicity, education level, gender). Importantly, we are restricted to the use of a composite depression and/or anxiety variable, as BC uses diagnostic code 50B to indicate depression, anxiety, or both. Therefore, we cannot tease apart these two conditions. There is also the possibility of misclassification of depression and/or anxiety given our reliance on diagnostic codes, but it is unlikely that misclassification would differ between groups and non-differential misclassification would represent a conservative bias. Additionally, some individuals in Symp No Endo may have had endometriosis that was not detected in their index surgery, and thus they may be misclassified as not having endometriosis. While using pathologically-confirmed endometriosis ensured a higher level of confidence in our endometriosis sample, this criteria may exclude some individuals who do not receive a pathological diagnosis (e.g. those who only receive a visual diagnosis at surgery) and may select for more severe endometriosis cases. Future research including individuals with visually and/or clinically diagnosed endometriosis would improve the generalizability of the

findings. This study would have benefited from a control group of people who were healthy controls, however this was not available in our data. Further, a direct measure of pain or a patient-reported pain variable would have improved this study. Finally, our sensitivity analyses include groups with small sample sizes, and thus must be interpreted with caution.

## Conclusions

In a large group of patients, pathologically-assessed for endometriosis, we find support for both the disease-focused and pain-focused hypotheses for the association between endometriosis and depression and/or anxiety. This work also supports the new model of endometriosis as a complex, systemic disease, rather than simply a gynecologic condition affecting the pelvis [16]. Previous research has shown that interdisciplinary care, where endometriosis is treated as a systemic condition, may improve patient outcomes [34]. Therefore, future work should aim to better understand each of these mechanisms independently and in combination, in order to optimize treatment options for patients. 

## Supplementary Information

Supplementary Material 1: Supplementary Table 1. Diagnostic codes for mental health disorders. Supplementary Table 2. Comparing characteristic of Symp Endo^a^ to Infertility/No Pain group. Supplementary Table 3. Unadjusted and adjusted hazard ratios for Symp Endo^a^ to Infertility/No Pain group.

## Data Availability

The datasets generated and/or analysed during the current study are not publicly available for privacy reasons but are available from PopData BC after application and data steward review and approval. The authors do not have permission to share the data, but other authors can apply for their own access to these datasets.

## Abbreviations

BC: British Columbia
SRI: Serotonin Reuptake Inhibitor
BO: Bilateral Oophorectomy
BSO: Bilateral Salpingoophorectomy
UO: Unilateral Oophorectomy
USO: Unilateral Salpingoophorectomy
HR: Hazard Ratio
CI: Confidence Interval

## References

[1] Vercellini P, Viganò P, Somigliana E, Fedele L. Endometriosis: pathogenesis and treatment. Nat Rev Endocrinol. 2014;10(5):261–75. 24366116 10.1038/nrendo.2013.255

[2] Bulletti C, Coccia ME, Battistoni S, Borini A. Endometriosis and infertility. J Assist Reprod Genet. 2010;27(8):441–7. 20574791 10.1007/s10815-010-9436-1 PMC2941592

[3] Parasar P, Ozcan P, Terry KL. Endometriosis: epidemiology, diagnosis and clinical management. Curr Obstet Gynecol Rep. 2017;6(1):34–41. 29276652 10.1007/s13669-017-0187-1 PMC5737931

[4] Bougie O, McClintock C, Pudwell J, Brogly SB, Velez MP. Long-term follow-up of endometriosis surgery in Ontario: a population-based cohort study. Am J Obstet Gynecol. 2021;225(3):270.e1-270.e19. 33894154 10.1016/j.ajog.2021.04.237

[5] Allaire C, Bedaiwy MA, Yong PJ. Diagnosis and management of endometriosis. Can Med Assoc J. 2023;195(10):E363-71. 36918177 10.1503/cmaj.220637 PMC10120420

[6] Gao M, Koupil I, Sjöqvist H, Karlsson H, Lalitkumar S, Dalman C, et al. Psychiatric comorbidity among women with endometriosis: nationwide cohort study in Sweden. Am J Obstet Gynecol. 2020;223(3):415.e1-415.e16. 32112731 10.1016/j.ajog.2020.02.033

[7] Friedl F, Riedl D, Fessler S, Wildt L, Walter M, Richter R, et al. Impact of endometriosis on quality of life, anxiety, and depression: an Austrian perspective. Arch Gynecol Obstet. 2015;292(6):1393–9. 26112356 10.1007/s00404-015-3789-8

[8] Warzecha D, Szymusik I, Wielgos M, Pietrzak B. The impact of endometriosis on the quality of life and the incidence of depression—a cohort study. Int J Environ Res Public Health. 2020;17(10):3641. 32455821 10.3390/ijerph17103641 PMC7277332

[9] Knoll AD, MacLennan RN. Prevalence and correlates of depression in Canada: findings from the Canadian community health survey. Canadian Psychology / Psychologie canadienne. 2017;58(2):116–23.

[10] Watterson RA, Williams JVA, Lavorato DH, Patten SB. Descriptive epidemiology of generalized anxiety disorder in Canada. Can J Psychiatry. 2017;62(1):24–9. 27310239 10.1177/0706743716645304 PMC5302105

[11] Škegro B, Bjedov S, Mikuš M, Mustač F, Lešin J, Matijević V, et al. Endometriosis, pain and mental health. Psychiatr Danub. 2021;33(Suppl 4):632–6. 34718292

[12] van Barneveld E, Manders J, van Osch FHM, van Poll M, Visser L, van Hanegem N, et al. Depression, anxiety, and correlating factors in endometriosis: a systematic review and meta-analysis. J Womens Health. 2022;31(2):219–30. 10.1089/jwh.2021.0021 34077695

[13] Facchin F, Barbara G, Saita E, Mosconi P, Roberto A, Fedele L, et al. Impact of endometriosis on quality of life and mental health: pelvic pain makes the difference. J Psychosom Obstet Gynaecol. 2015;36(4):135–41. 26328618 10.3109/0167482X.2015.1074173

[14] Eriksen HLF, Gunnersen KF, Sørensen JA, Munk T, Nielsen T, Knudsen UB. Psychological aspects of endometriosis: differences between patients with or without pain on four psychological variables. Eur J Obstet Gynecol Reprod Biol. 2008;139(1):100–5. 18022311 10.1016/j.ejogrb.2007.10.002

[15] Li T, Mamillapalli R, Ding S, Chang H, Liu ZW, Gao XB, et al. Endometriosis alters brain electrophysiology, gene expression and increases pain sensitization, anxiety, and depression in female mice†. Biol Reprod. 2018;99(2):349–59. 29425272 10.1093/biolre/ioy035 PMC6692844

[16] Taylor HS, Kotlyar AM, Flores VA. Endometriosis is a chronic systemic disease: clinical challenges and novel innovations. Lancet. 2021;397(10276):839–52. 33640070 10.1016/S0140-6736(21)00389-5

[17] British Columbia Ministry of Health. : Medical Services Plan (MSP) Payment Information File. V2. Population Data BC. Data Extract. MOH(2021). https://www.popdata.bc.ca/data/health/msp

[18] British Columbia Ministry of Health. Consolidation File (MSP Registration & Premium Billing). V2. Population Data BC. Data Extract. MOH(2021). https://www.popdata.bc.ca/data/health/msp

[19] British Columbia Ministry of Health. Vital Events Births. V2. Population Data BC. Data Extract. MOH(2021). https://www.popdata.bc.ca/data

[20] British Columbia Ministry of Health. Vital Events Deaths. V2. Population Data BC. Data Extract. MOH(2021). https://www.popdata.bc.ca/data

[21] British Columbia Ministry of Health. Vital Events Stillbirths. V2. Population Data BC. Data Extract. MOH(2021). https://www.popdata.bc.ca/data

[22] Canadian Institute for Health Information. Canadian Institute for Health Information. Discharge Abstract Database (Hospital Separations). V2. Population Data BC. Data Extract. MOH(2021). https://www.popdata.bc.ca/data/health/dad

[23] BC Cancer: BC Cancer Registry Data. V2. Population Data BC. Data Extract. BC Cancer(2021). https://www.popdata.bc.ca/data/health/bccancer

[24] British Columbia Ministry of Health: PharmaNet. V2. Population Data BC. Data Extract. Data Stewardship Committee(2021). https://www.popdata.bc.ca/data/health/pharmanet

[25] Juurlink D, Preyra C, Croxford R, Chong A, Austin P, Tu J et al. Canadian Institute for health information discharge abstract database: A validation study. Toronto, ON: Institute for clinical evaluative sciences;2006. Inst Clin Eval Sci.2006; 1–77.

[26] Kisely S, Lin E, Lesage A, Gilbert C, Smith M, Campbell LA, et al. Use of administrative data for the surveillance of mental disorders in 5 provinces. Can J Psychiatry. 2009;54(8):571–5. 19726010 10.1177/070674370905400810

[27] Hanley GE, Morgan S. On the validity of area-based income measures to proxy household income. BMC Health Serv Res. 2008;8(1):79. 18402681 10.1186/1472-6963-8-79 PMC2358887

[28] Giannella L, Marconi C, Di Giuseppe J, Delli Carpini G, Fichera M, Grelloni C, et al. Malignant transformation of postmenopausal endometriosis: a systematic review of the literature. Cancers (Basel). 2021;13(16):4026. 34439184 10.3390/cancers13164026 PMC8394809

[29] Streuli I, Gaitzsch H, Wenger JM, Petignat P. Endometriosis after menopause: physiopathology and management of an uncommon condition. Climacteric. 2017;20(2):138–43. 28286987 10.1080/13697137.2017.1284781

[30] Inceboz U. Endometriosis after menopause. Womens Health. 2015;11(5):711–5. 10.2217/whe.15.59 26343168

[31] Dickens C, McGowan L, Clark-Carter D, Creed F. Depression in rheumatoid arthritis: a systematic review of the literature with meta-analysis. Psychosom Med. 2002;64(1):52–60. 11818586 10.1097/00006842-200201000-00008

[32] Jones JL, Nguyen GC, Benchimol EI, Bernstein CN, Bitton A, Kaplan GG, et al. The impact of inflammatory bowel disease in Canada 2018: quality of life. J Can Assoc Gastroenterol. 2019;2(Supplement1):S42–8. 31294384 10.1093/jcag/gwy048 PMC6512247

[33] Irving P, Barrett K, Nijher M, de Lusignan S. Prevalence of depression and anxiety in people with inflammatory bowel disease and associated healthcare use: population-based cohort study. Evid Based Ment Health. 2021;24(3):102–9. 33785498 10.1136/ebmental-2020-300223 PMC8311072

[34] Allaire C, Long AJ, Bedaiwy MA, Yong PJ. Interdisciplinary teams in endometriosis care. Semin Reprod Med. 2020;38(2–03):227–34. 33080631 10.1055/s-0040-1718943

