# Supplementary material for: Endometriosis, symptoms, and risk for depression and/or anxiety: a population-based retrospective study
Source: BMC Womens Health. 2025 Oct 8;25:479. doi: 10.1186/s12905-025-04022-5 (PMC12509389; doi:10.1186/s12905-025-04022-5)
Supplement: Supplementary file 1 — Supplementary Material 1: Supplementary Table 1. Diagnostic codes for mental health disorders. Supplementary Table 2. Comparing characteristic of Symp Endoa to Infertility/No Pain group. Supplementary Table 3. Unadjusted and adjusted hazard ratios for Symp Endoa to Infertility/No Pain group. [file 12905_2025_4022_MOESM1_ESM.docx]

Supplementary Table 1**:** Diagnostic codes for mental health disorders

| Diagnosis group | ICD-10-CA | ICD-9 |
| --- | --- | --- |
| **Mental Health Condition** | | |
| Depression | F32-39 | 296.2-296.36; 311; 50B* |
| Anxiety disorder | F40-43 | 300; 50B* |
| **Mental Health Disorders Controlled For** | | |
| Schizophrenia | F20-29 | 295 |
| Bipolar disorder | F30-31 | 296.0-296.16; 296.4-296.9 |
| Personality disorder | F60-69 | 301 |

*code is specific to British Columbia

Supplementary Table 2. Comparing characteristic of Symp Endo^a^ to Infertility/No Pain group.

| **Characteristics** | **Symp Endo^a^ (Symptomatic Endometriosis,**  **N=1866)** | **Infertility/No Pain (N=174)** | **P-value** | **Effect Size** |
| --- | --- | --- | --- | --- |
| Age (Mean, SD) | 35.2 (7.34) | 34.2 (4.06) | 0.006 | 0.12 |
| SES Quintile |  |  |  |  |
| 1 | 383 (20.5%) | 42 (24.1%) | 0.623 | 0.01 |
| 2 | 392 (21.0%) | 31 (17.8%) |  |  |
| 3 | 357 (19.1%) | 30 (17.2%) |  |  |
| 4 | 372 (19.9%) | 39 (22.4%) |  |  |
| 5 | 359 (19.2%) | 32 (18.4%) |  |  |
| Missing | ≤ 5 | 0 (0%) |  |  |
| Year of pathology report |  |  |  |  |
| Median [Min, Max] | 2004 [2000, 2008] | 2004 [2000, 2008] | 0.54 | 0.04 |
| Premenopausal age at surgery (proxy using age < 50) | 2355 (100%) | 174 (100%) | NA | NA |
| Postmenopausal age at surgery (proxy using age >= 50) | 0 (0%) | 0 (0%) | NA | NA |
| **Procedures performed during the index surgery** |  |  |  |  |
| Hysterectomy | 0 (0%) | 0 (0%) | NA | NA |
| Salpingectomy | 34 (1.8%) | ≤ 5 | 1 | 0.000 |
| UO/USO | 313 (16.8%) | ≤ 5 | <0.001 | 0.12 |
| BO/BSO/second UO/USO | 45 (2.4%) | 0 (0%) | 0.072 | 0.04 |
| Biopsy | 191 (10.2%) | 19 (10.9%) | 0.878 | 0.003 |
| Adhesiolysis | 498 (26.7%) | 44 (25.3%) | 0.756 | 0.007 |
| Excision | 653 (35.0%) | 88 (50.6%) | <0.001 | 0.09 |
| Ablation | 355 (19.0%) | 63 (36.2%) | <0.001 | 0.12 |
| Other | 898 (48.1%) | 104 (59.8%) | 0.004 | 0.06 |
| **Surgical approach for index surgery** |  |  |  |  |
| Laparoscopic | 553 (29.6%) | 66 (37.9%) | 0.029 | 0.05 |
| Abdominal | 223 (12.0%) | 6 (3.4%) | 0.001 | 0.07 |
| Vaginal | 163 (8.7%) | 21 (12.1%) | 0.184 | 0.03 |
| Laparoscopic/vaginal combination | 90 (4.8%) | 11 (6.3%) | 0.491 | 0.02 |
| Missing | 243 (13.0%) | 10 (5.7%) | 0.008 | 0.06 |
| **Clinical Indication for surgery from pathology report** |  |  |  |  |
| Mass/Cancer | 273 (14.6%) | ≤ 5 | <0.001 | 0.10 |
| Pain | 335 (18.0%) | 0 (0%) | <0.001 | 0.13 |
| Endometrioma | 277 (14.8%) | 22 (12.6%) | 0.501 | 0.01 |
| Other Endometriosis | 644 (34.5%) | 84 (48.3%) | <0.001 | 0.08 |
| Infertility | 85 (4.6%) | 174 (100%) | <0.001 | 0.80 |
| Cyst | 371 (19.9%) | 27 (15.5%) | 0.197 | 0.03 |
| Adenomyosis | 0 (0%) | 0 (0%) | NA | NA |
| Fibroids | 55 (2.9%) | 7 (4.0%) | 0.576 | 0.01 |
| **Surgical history** |  |  |  |  |
| Prior BO/BSO, second UO/USO | ≤ 5 | 0 (0%) | 1 | 0.000 |
| Endometriosis surgery >45 days before path report | 254 (13.6%) | 7 (4.0%) | <0.001 | 0.08 |
| **Pathologically diagnosed conditions** |  |  |  |  |
| Endometriosis | 1866 (100%) | 153 (87.9%) | <0.001 | 0.33 |
| Endometrioma/  endometriotic cyst | 807 (43.2%) | 44 (25.3%) | <0.001 | 0.10 |
| Endometriosis in ovary - non-endometrioma/ endometriotic cyst | 230 (12.3%) | 10 (5.7%) | 0.014 | 0.05 |
| Other endometriosis in the pelvis | 1127 (60.4%) | 108 (62.1%) | 0.726 | 0.01 |
| Other endometriosis outside the pelvis | 79 (4.2%) | ≤ 5 | 0.159 | 0.03 |
| Endometrial Hyperplasia | 8 (0.4%) | ≤ 5 | 0.463 | 0.02 |
| Adenomyosis | 31 (1.7%) | ≤ 5 | 0.843 | 0.004 |
| Fibroids | 95 (5.1%) | 7 (4.0%) | 0.663 | 0.01 |

Abbreviations: SD, Standard Deviation; UO, Unilateral oophorectomy; USO, Unilateral Salpingoophorectomy; BO, Bilateral Oophorectomy; BSO, Bilateral Salpingoophorectomy.

^a^ Group criteria: Symp Endo had pathologically-confirmed endometriosis and symptoms before index surgery. We removed individuals above age 50 and those who had a hysterectomy at the index surgery from this group, as they could not be experiencing infertility.

Supplementary Table 3. Unadjusted and adjusted hazard ratios for Symp Endo^a^ to Infertility/No Pain group.

|  | Symp Endo^a^ (Symptomatic with Endometriosis) | Infertility/No Pain |
| --- | --- | --- |
| N | 1866 | 174 |
| Mean Follow-up Time (years) | 8.65 | 9.50 |
| Number of Events | 726 | 51 |
| Crude hazard ratio (95% CI) | Ref | 0.75 (0.57-1.00) |
| Adjusted hazard ratio^b^ (95% CI) | Ref | 0.74 (0.55-0.98) |

*HR, Hazard Ratio; CI, Confidence Interval.*

^a^ Group criteria: Symp Endo had pathologically-confirmed endometriosis and symptoms before index surgery. We removed individuals above age 50 and those who had a hysterectomy at the index surgery from this group, as they could not be experiencing infertility.

*^b^ Variables adjusted for: Age, income quintile, and other diagnoses from index surgery pathology report (endometrial hyperplasia, adenomyosis, or fibroids).*
